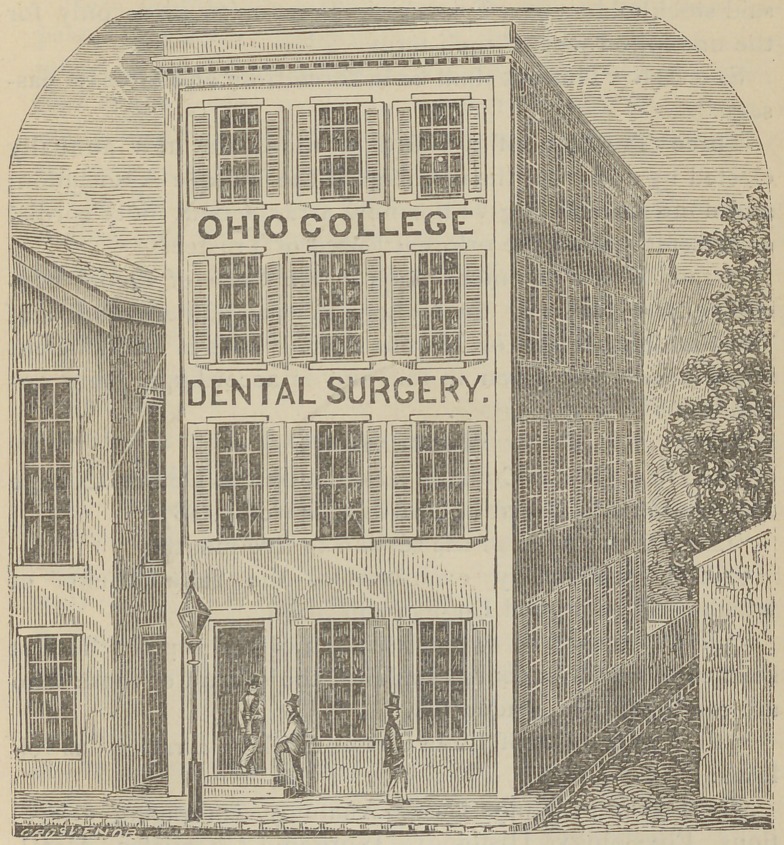# Historical Sketch of the Ohio College of Dental Surgery

**Published:** 1879-05

**Authors:** Geo. Watt


### THE DENTAL REGISTER.

VOL. XXXIII.] **MAY, 1879.** No 5.

### HISTORICAL SKETCH OF THE OHIO COLLEGE OF DENTAL SURGERY.

*Prepared for the First Annual Meeting of the Alumni Association.*

BY GEO. WATT. M. D., D. D. S.

That the influence of a College devoted to Dental Surgery, may be du'y appreciated, it is necessary to glance at the attainments of human civilization, in the direction of dental science, previous to the establishment of such a College. A very slight observation in this direction will develop the fact, that ages on ages of dental distress were necessary to enlighten the human mind, so as to enable it to demand this instrumentality in alleviation. Within the memory of the writer the dentist was on the same platform with the traveling tinker, who, trudging the highways and byways, turned aside to mend the kettles and candlesticks of the adjacent farm houses. And the dentists of this description were such a decided improvement on the blacksmith, the butcher and the barber—if not the physician and surgeon—who hitherto had the care of the teeth, that the demand for their services increased, till their circuits became shorter, their movements slower, till, in obedience to the law of supply and demand, they gravitated to a central point, and a new thing under the sun was revealed, in the shape of resident dentists.

When men began to give their whole time to the care of the teeth, new thoughts were developed, till, by the light of the knowledge thus acquired, they were able to see "the darkness of ignorance," which pervaded the race, in reference to these important organs. A sight of our wants is the first step towards their supply. The tinker developed into a traveling dentist, he to a resident dentist, he to a seeker, that is to a dental student, he to an imparter, otherwise a dental teacher, he to a co-operator with other men of science; and, co-operation in the cultivation of various sciences means a college; and, with dental science as the leading thought, we have a dental college; and all this in obedience to the law of supply and demand.

A plain boy in a picture, admiring the costume of his dandy comrade, exclaims, "Why gracious, Jim, what a tie!" And the reply is, "Yes, Fred, that is rather exquisite; but, you see, I give my whole mind to it." Giving the whole mind to it, explains the success in dental surgery, as it does in the neck-tie. The surgeon dentist of the early period must divide his attention, or travel. How fortunate for humanity that he chose the latter, and thereby taught the advantages of, and the necessity for, a higher order of attainment than that possessed by the knight of the torturing turnkey! And how different his character and calling from those of the traveling quack of to-day, whose propensity to travel should be promptly promoted by pedal propulsion, as the doors are closed in his brazen face.

That the character and career of the early dentists are not overdrawn, can be attested by some of our most eminent fathers and brethren yet living. The lack of privilege in this early day, and the great struggle against fearful odds necessary to succss, are vividly impressed on the mind of the writer, by events occurring in the experience of two of our profession, still with us, who have a more extended experience in teaching practical dentistry than any other men living. In September, 1841, one of them and the writer left college, agreeing to keep up a regular correspondence. The writer began the study of medicine, and soon after received a letter from his comrade, stating the fact that he was teaching and studying, adding, "The first thing you know, Mister, I'll be a real, genuine, scientific dentist." That's the doctrine, thought I, scientific or nothing. But how is it to be done? Before myself, as a medical student, I could see educated private preceptors, libraries, colleges, hospitals, universities, medical societies and periodicals; but what of opportunity and privilege could I see before the dental student too earnest and conscientious to be other than scientific? But he says he'll be a "scientific dentist," and he will. But how? You who have listened to his lectures, who have read the DENTAL REGISTER, who have studied the various editions of *Taft's Operative Dentistry*, tell us.

The other event, occurring a few years earlier, but within the writers's memory, relates to one who, in a certain sense, is "the father of us all." Determined to stand or fall with dental science, notwithstanding his medical education, in the peregrinations necessary then to such a career, weary, ill and hungry, with a horse as hungry as himself, he approached an Ohio village. A young lawyer, possessing both a heart and a soul, invited him to his father's house, thus rendering it unnecessary for him to reveal his penniless condition, and secured for him a number of remunerating patients among his acquaintances. But the fools were not all dead then; and it is to be feared the race is not even yet extinct. When about ready to leave, a man, who had had an artificial tooth inserted, got out a warrant for the arrest of the dentist, for obtaining money on false pretenses, after finding that the new tooth was no better than a natural one. The young lawyer was still equal to the emergency, and again sent the dentist to his father's house, keeping him wholly ignorant of the existence of the warrant, reporting to the constable that he was gone, as he truly was, but not far. Now, if from any or all of these trying circumstances, this young dentist had become discouraged, and had abandoned the practice of dentistry, what about the history of the Ohio College of Dental Surgery? For this talk is all about one of its incorporators, its principal stockholder, the President of its Board of Trustees, its oldest teacher, our professional father, James Taylor.

It was "in the fullness of time" that God sent forth his Son; and the fullness of time is ever the crisis in Providence and in science. When humanity is ready for a new discovery, or a new era in progress, it makes its appearance, seeming not to emanate from individual mind, so much as from the combined thought of the race. Dental colleges accord with no new rule in regard to human progress; but the thought was ripe in the minds of those giving their entire professional attention to the mouth and its adjacent organs. This thought assumed practical shape first in the state of Maryland, resulting in the establishment of the "Baltimore College of Dental Surgery." But the dentists of the West, though fewer in number, and more widely dispersed, were equally ripe for action; and this action promptly asserted itself in the organization of our *Alma Mater*, "The Ohio College of Dental Surgery."

The charter or "act" of the legislature of Ohio, by which the insttiution came into legal existence, is as follows:

"AN ACT

"*To Authorize the Establishment of a College of Dental Surgery.*

"*Section* 1. *Be it enacted by the General Assembly of the State of Ohio*, That B. P. Aydelotte, Robert Buchanan, Dr. Israel M. Dodge, Wm. Johnson, J. P. Cornell and Calvin Fletcher, of Cincinnati, Dr. G. S. Hampstead, of Portsmouth. and Dr. Samuel Martin, of Xenia, and their successors, are hereby constituted and appointed a Board of Trustees, with power to establish a College of Dental Surgery in the city of Cincinnati, and said Board is hereby declared to be a body corporate and politic, with perpetual succession, and shall be known by the name and style of the Trustees of the Ohio College of Dental Surgery; and said Board shall have power to acquire, hold and convey property for the endowment of said College, to sue and be sued, contract and be contracted with, plead and be impleaded, defend and be defended, answer and be answered unto, in all courts and places, and in all matter and causes whatsoever; provided that no part of the estate, either real or personal, which said corporation may at any time acquire, shall be employed in the business of banking, or for any other purpose than that designated by this act; and provided, also, that the revenues arising from the property which said incorporation shall be permitted to hold, for the purposes above specified, shall not exceed five thousand dollars per annum.

"*Sec.* 2. That the said incorporation may have a common seal, which may be altered, broken or renewed at pleasure.

"*Sec.* 3. That the officers of said incorporation shall be a President, Vice-President, Registrar and Treasurer, who shall be elected annually, by said Board of Trustees, at such time, and in such manner as the said Board may direct, and shall hold their offices till their successors are chosen.

"*Sec.* 4. That the Trustees of said incorporation shall have full power to create and establish such professorships as they may deem necessary for said College, and that they may, at any time, appoint or dismiss all such professors or lecturers as they may think proper; also, to make and ordain such by-laws, rules and regulations as they may deem necessary for the government and well being of said College; provided such by-laws, rules and regulations are not inconsistent with the Constitution and laws of this State and the United States; and, provided also, that no branches of medical science shall be taught except those necessary to dental surgery.

"*Sec.* 5. That all vacancies which may occur from death, resignation or otherwise, in the Board of Trustees of the aforesaid incorporation, shall be filled by the remaining members of said Board.

"*Sec.* 6. That said Board of Trustees shall have power, and are hereby authorized to confer the degree of Doctor of Dental Surgery, and grant diplomas for the same, under the seal of the incorporation; provided that no diploma thus granted shall confer any privilege further than the practice of dental surgery.

"*Sec.* 7. That the said corporation shall be subject to all the regulations and liabilities of an act instituting proceedings against corporations not possessing banking powers, and to provide for the regulations of corporations generally, passed March 7, 1842.

"*Sec.* 8. This act shall take effect from and after its passage.

"JOHN M. GALLAGHER,
"Speaker of the House of Representatives.
"DAVID CHAMBERS,
"January 21, 1845. "Speaker of the Senate."

In the spring of 1845 the Trustees appointed by this act met and organized, by the appointment of B. P. Aydelotte, M. D., D. D., President, and Israel M. Dodge, M. D., Secretary; and then organized the Ohio College of Dental Surgery by the creation of the following departments, viz.:

Dental Anatomy and Physiology, of which Jesse W. Cook, M. D., D. D. S., was made professor.

Dental Pathology and Therapeutics, of which Melancthon Rogers, M. D., D. D. S., was elected professor.

Practical Dentistry and Pharmacy, of which James Taylor, M. D., D. D. S., was appointed professor.

Jesse P. Judkins, M. D., was appointed Demonstrator of Anatomy; and Professor Taylor agreed, for the present, to discharge the duties of Demonstrator of Practical Dentistry.

The Faculty elected Prof. Cook, Dean; and he issued the first annual announcement, and the College session, for its first course of lectures, opened on the first Monday of November, 1845, and closed, on or about the 20th day of February, 1846, four young men receiving degrees, two of whom are yet alive, and in active practice. President Aydelotte delivered the opening address, conferred the degrees, and, in behalf of the College, gave each graduate a copy of the Holy Bible, (a custom which has been observed ever since). Prof. Cook gave the valedictory address to the graduates. And thus ended the first voyage of our *Alma Mater* on the sea of science.

But time and strength will not permit a similarly minute account of her subsequent voyages, nor is such even desirable, as each class has its own historian. Only a general review will now be presented.

For the second session the venerable Christian philosopher, Elijah Slack, D. D., LL. D., was appointed Lecturer on Chemistry; and, it is believed, delivered the first course of lectures, on this science, ever given to dental students.

In 1847, Prof. Cook resigned his chair; and the Trustees filled it by electing J. F. Potter, M. D., and the Faculty appointed Dr. Wm. M. Hunter, Demonstrator of Mechanical Dentistry.

In 1848, Profs. Rogers and Potter resigned; and George Mendenhall, M. D., was elected Professor of Dental Pathology and Therapeutics, and John T. Shotwell, M. D., Professor of Anatomy and Physiology. The Faculty appointed A. M. Leslie, D. D. S., Demonstrator of Mechanical Dentistry, and Charles H. Raymond, Lecturer on Chemistry.

In the department of Anatomy, Prof. Shotwell was succeeded by Thomas Wood, M. D.; he by C. B. Chapman, M. D.; he by Charles Kearns, M. D.; he by Wm. Clendenin, M. D. The character and standing of the professors elected to teach this science, show the high estimate placed upon it by the Trustees and stockholders of the College.

In 1850, a Professorship of Mechanical Dentistry was created, and A. M. Leslie, D. D. S., was elected to the new chair, which place has since been held by John Allen, D. D. S., H. R. Smith, D. D. S., M. D., Joseph Richardson, M. D., D. D. S., C. M. Wright, D. D. S., J. A. Watling, D. D. S., Wm. Van Antwerp, D. D. S., M. D., N. S. Hoff, D. D. S., and J. R. Clayton, D. D. S., whom to name is to eulogize our *Alma Mater.*

The department of Chemistry struggled for existence. After Dr. Raymond, G. L. Van Emon, D. D. S., was appointed lecturer, in 1851. And in 1853, Geo. Watt, M. D., succeeded him as lecturer, and he was, in turn, succeeded by George M. Kellogg, M. D.; in 1855, the science was regarded as worthy of a professorship, a new chair was created, called "Chemistry and Metallurgy," and Geo Watt, M. D., D. D. S., was elected to fill it. The position has since been filled by H. A. Smith, D. D. S., S. P. Cutler, D. D. S., J. G. Willis, M. D., D. D. S. (?), and J. S. Cassidy, M. D., D. D. S., who is the present incumbent.

The chair of Pathology, after the resignation of Prof. Mendenhall, was filled by the election of J. B. Smith, M. D.; and this position has been subsequently held by Geo. Watt, M. D., Edward Rives, M. D.. F. Brunning, M. D., and A. O. Rawls, D. D. S., the present incumbent.

In 1851, a chair of Operative and Mechanical Dentistry was created, and John Allen, D. D. S., was elected to fill it. In 1853 this was divided, leaving the department of Operative Dentistry to Professor Allen, who in 1854 resigned the chair, and was succeeded by Jonathan Taft, D. D. S., who occupied the place till March, 1878.

A chair of Clinical Dentistry was established (at a date not now recollected), and was filled at various times by W. T. Arrington, D. D. S., J. A. Watling, D. D. S., C. R. Butler, D. D. S., Wm. Taft, D. D. S., M. D., H. M. Reid, D. D. S., J. I. Taylor, D. D. S., and H. A. Smith, D. D. S., the present incumbent.

Additional studies, other than those indicated by the names, were added to most, if not all the departments, such as Dental Hygiene, Microscopy, Histology, Metallurgy, Materia Medica, etc., and special professorships were, from time to time, provided for the departments of Oral Surgery, Irregularities, etc. And besides these, special clinical instructors have been selected for many years, from among those in the dental profession of high repute as operators. It is probable that our College was the pioneer in this direction; but, at any rate, the example has been well and profitably followed.

In 1850 the Faculty adopted the following resolution, which was continued in force by the College Association:

"*Resolved*, That a committee of two from the medical, and three from the dental profession be selected, annually, to examine, in connection with the Faculty, the candidates for graduation."

After a fair experiment, this was rescinded, in 1860, having been found unprofitable, and tending to lower, rather than to elevate the character of the examinations, as it was found that a number of candidates received degrees who would have been rejected by a vote of the faculty alone.

Previous to the session of 1851 the duties of the College were discharged in a building leased for the purpose. True, it had been mainly built by the distinguished educator, John L. Talbot, with special reference to the wants of this College The lease, for ten years, included the privilege of purchase. By correspondence and personal solicitation, arrangements were made to buy the building, shares of stock having been issued, were promptly taken by members of the profession, and a few others, interested in dental education. It would be unjust should we fail to give Professor Taylor due credit for this effort. Accordingly, in November, 1851, the college session was opened in a building owned by the profession, and specially dedicated, for all time, to the cause of dental education, which was another new thing under the sun.

The stockholders held their first regular meeting in the lecture room of the college, February 19, 1852. Dr. Charles Bonsall was called to the chair, and Dr. Thomas Wood was appointed Secretary. Drs. Thos. Wood, H. R. Smith and James Taylor, were appointed to report a draft of a constitution, which, after some modifications, was adopted as follows:

PREAMBLE.

The stockholders and alumni of the Ohio College of Dental Surgery believing that the interests of dental science require a more thorough course of dental instruction than has heretofore usually been afforded, and that this can be best accomplished by institutions devoted expressly to this object, and that associations entered into with the proper spirit must afford increased facilities for our mutual improvement, and for the promotion of dental science, and that to further the views of those who have already engaged in the enterprise of permanently founding the Ohio College of Dental Surgery; therefore, for the promotion of these objects, and all such others as may conduce to the advance of our science, we adopt the following

### CONSTITUTION.

ARTICLE I. This society shall be called the Ohio Dental College Association.

ARTICLE II. The officers of this association shall consist of a president, two vice-presidents, a secretary, a treasurer, and an examining committee of five; three from the dental, and two from the medical profession, who shall be chosen by ballot at each annual meeting of the association, and who shall perform such duties as usually pertain to their respective offices.

ARTICLE III. The members of this association shall consist of two classes: 1. The holders of stock in the Ohio Dental College; 2. All graduates of the institution may become members on receiving a vote of two-thirds of the members present, signing the constitution, and obligating themselves to pay annually into the treasury a sum equal to the interest on one share of stock.

ARTICLE IV. Any member may be expelled by a vote of two-thirds of the members present, for immoral or unprofessional conduct.

*Section* 1. No expelled member shall have any of his annual contributions refunded, but if he is a stockholder, he may sell his stock, always giving the association the first privilege as purchaser.

*Section* 2. Stock may be sold or transferred, but the purchaser shall not be entitled to membership except by a vote of two-thirds of the members present at any annual meeting.

*Section* 3. The purchase of a share of stock shall not entitle the holder to membership unless he shall receive a vote of two-thirds of the members present.

ARTICLE V. The meetings of the association shall be held annually, in the Ohio Dental College in Cincinnati, at ten o'clock a. m., of the day preceeding the annual commencement; and the president may call a meeting when requested by five members; and in all meetings it shall require thirteen to form a quorum.

ARTICLE VI. In all matters relating to the property held by the association, each stockholder shall have as many votes as he may have shares of stock, and in case of unavoidable absence he may vote by proxy.

ARTICLE VII. This constitution may be altered or amended by a vote of two-thirds present at any annual meeting, except such change as would affect the shares of stock; which amendment must be proposed at one annual meeting, and acted on at the next.

The first election of officers resulted in the selection of James Taylor, President; W. M. Wright, First Vice-President; Thos. Wood, Second Vice-President; Chas. Bonsall, Secretary; Edward Taylor, Treasurer. And thus was the Ohio Dental College Association organized, and equipped for action; and it has had virtual control of the college ever since, in its educational, as well as in its financial aspects. Eighteen members were present, and signed the constitution, of whom, I believe, fourteen are still spared to us. Eleven absent stockholders were represented by proxy.

At this first meeting the stockholders generously relinquished their interest on stock, for the good of the college, for three years; and this principle of generosity, has ruled ever since. New shares of stock were issued and taken; and the present list of stockholders is as follows:

James Taylor, D. W. Clancey, Chas. Bonsall, A. Berry, Thos. Wood, Geo. Mendenhall, C. R. Taft, J. M. Brown, Sam'l Wardle, H. R. Smith, J. Taft, H. A. Smith, Jas, Leslie, B. D. Wheeler, J. G. Cameron, F. H. Hunter, H. M. Reid, J. I. Taylor, N. S. Hoff, E. G. Betty, C. H. Rosenthal, Wm. Taft, Cincinnati, O.; C. W. Spalding, H. E. Peebles, H. J. McKellops, Isaiah Forbes, Aaron Blake, Henry Barron, A. Sloan, A. M. Leslie, Wm. N. Morrison, H. S. Chase, St. Louis, Mo.; Geo. H. Cushing, Chicago, Ills.; I. B. Branch, Galena, Ills,; J. M. Lewis, Marion, Ills,; W. W. Allport, J. D. Quinlan, Jno. C. Fuller, Emanuel Honsinger, Wm. Albaugh, Jas. C. Dean, Chicago, Ills.; W. P. Horton, Cleveland, O. Wm. M. Wright, Pittsburg, Pa.; S. S. White, Philadelphia, Pa.; J. B. Dunlevy, Pittsburg. Pa., A. B. Robbins, Meadville, Pa.; John Allen, New York, N. Y.; L. D. Walter, Rochester, N. Y.; Geo. E. Hayes, Buffalo, N. Y.; Geo. Watt, Xenia, O.; A. S. Talbert, Lexington, Ky.; D. Daugherty, Bardstown, Ky.; J. W. Baxter, Warsaw, Ky.; J. A. McClelland, W. G. Redman, Louisville, Ky.; Jas. S. Knapp, Chas. E. Kells, W. S. Chandler, G. S. Fredericks, New Orleans, La.; M. M. Manlove, Logansport, Ind.; J. P. Ulrey, Rising Sun, Ind. E. Bray, Evansville, Ind.; Wm. R. Webster, Richmond, Ind.; W. F.

Morrill, New Albany, Ind.; S. M. Cummins, Elkhart, Ind.; J. S. Rice, J. R. Clayton, Shelbyville, Ind.; E. A. Herman S. J. Cobb, W. H. Morgan, Nashville, Tenn.; J. T. McMillan, Paris, Ky.; Geo. W. Keely, Oxford, O.; D. Phillipps, Springfield, O.; S. M. Rhoads, Wooster, O.; E. C. Sloan, Ironton, O.; D. C. Whaley, Pomeroy, O.; J. B. Miner, David W. Perkins, Milwaukee, Wis.; Eli Collins, Little Rock, Ark.; N. B. Slayton, Florence, Italy, W. W. Sheffield, New London, Conn.; G. L VanEmon, Huntsville, Ala.; B. O. Doyle, J. F. Canine, Louisville, Ky.; A. O. Rawles, Lexington, Ky.; B. Corson, Middletown, Ohio; R. L. Evans, Toledo, Ohio; I. Williams, New Philadelphia, Ohio; Chas Welch, I. B. Welch, Wilmington, Ohio; W. H. Sedgwick, Granville, Ohio; C. E. Terry, Zurich, Switzerland; F. K. Jamison, Connersville, Ind.; A. F. Emminger, Columbus, Ohio.

In 1854, the old building, purchased from Mr. Talbot, having been found inadequate to the growing wants of the college, the stockholders took steps toward the erection of an entirely new edifice. As the location, College street, between Sixth and Seventh streets, was central, it was decided to rebuild on the same ground. With marvelous energy and promptness the new building was erected and furnished in time for the opening of the ensuing course of lectures. This is the first building erected for the sole and special purpose of dental education; and here is a general view of its outward appearance.

In 1865, a change in the charter and general management of the college occurred. Progress has ever been, and still is, the watchword of our *Alma Mater*. One object of the change was to bring the institution more directly under the immediate supervision and control of the College Association. A new act was passed by the legislature as follows:

A BILL TO REGULATE COLLEGES OF DENTAL SURGERY.

SECTION I. "*Be it enacted by the General Assembly of the State of Ohio*, That the Board of Trustees of any College of Dental Surgery heretofore incorporated and organized under any law of this State shall consist of nine members, and shall be elected by the stockholders of such College in a manner hereinafter provided.

SEC. II. "The stockholders of such College of Dental Surgery shall, at their first annual meeting after the passage of this act, proceed to elect nine Trustees of such College, three

OHIO COLLEGE

DENTAL SURGERY.

GROSVENOR

of whom shall serve as such for two years, and three of whom to serve as such for three years, and annually thereafter shall elect three Trustees to serve for the term of three years; *Provided*, that vacancies in said Board of Trustees, occurring from any other cause than expiration of the time of the Trustee creating such vacancy shall be filled by the election by said stockholders of a Trustee or Trustees to serve only for the unexpired part of such term.

SEC. III. "This act shall be in force from and after its passage."

Three Trustees, of a board of nine, are now annually elected by and from the members of the College Association; and none seemed to give the new arrangement more cordial endorsement than the original Board of Trustees.

A radical and advanced step, in the cause of dental education, was taken by the College Association and Board of Trustees, on the 5th of March, 1867. This is of sufficient importance to be given in full, and is accordingly here appended:

"REGULATIONS

of the Ohio Dental College, adopted by the Dental College Association and Board of Trustees, March 5, 1875.

"1st. An extension of the session to five months.

"2d. A preliminary examination, the requirements of which shall be a good English education.

"3d. There shall be two classes, junior and senior; the first shall consist of first course students, the second of those who are candidates for graduation.

"4th. The studies of these classes shall be arranged as follows:

"First year or junior class—Anatomy, embracing dissections, Physiology, Histology, Inorganic Chemistry, Metallurgy and Mechanical Dentistry

"Second year or senior class—Histology, Pathology, Dissections, Organic Chemistry, Therapeutics, Operative Dentistry and Dental Hygiene.

"5th. Members of the junior class will be required to pass an examination on the branches studied before entering the senior class. This may be at the close of the junior or the beginning of the senior course, at the option of the student. When this examination is satisfactory, a certificate of the fact, bearing the seal of the college, shall be given to the student, which shall entitle him to enter the senior class.

"6th. Applicants for admission to the senior class must pass a satisfactory examination of the junior course, except when, in special cases, the faculty may allow them to take a part of the junior course in connection with the senior, in which case this part of their examination will be deferred till the close of the senior term."

The division of the course with "junior" and "senior" studies, and the requirement in the first clause of the fifth section, viz: "Members of the junior class will be required to pass an examination on the branches studied before entering the senior class," were at this time, probably, new features in collegiate study. True, a leading University, after adopting similar measures, at a later date, claimed to be the pioneer, probably having overlooked the action of our ever wide-awake *Alma Mater*.

The influence of this College on the dental profession, and on society in general, can never be over-estimated. It is not claiming too much when we state that her *Alumni* have done their full share of solid thinking for our profession, especially in the last thirty years. They have furnished leading text books, leading writers for the periodical press, leading speakers and thinkers in the dental associations, leading investigators and experimenters, while they have not fallen behind any in collateral science and social qualities. It will be noticed, at a glance, that the professorships in our *Alma Mater*, through all the changes made necessary by time and circumstance, have been mainly held by her own *Alumni*, except where it was thought best to fill certain special chairs, from the medical profession. She always knew where to find the men she needed, and the thoroughness of her teachings rendered it quite unnecessary to go beyond the pale of her own family. Other dental schools also found in the ranks of her sons, the teachers wanted for their new institutions.

It is not the intention of this paper to notice the names, members, or the individual or special attainments of the several classes. To do this is probably the special province of the class historians. At any rate, it would be well that one individual of each class, self-appointed or otherwise, should see that a correct roll of his class is furnished for publication. In the partial lists, as now published, some names are inadvertently omitted, some are spelled incorrectly, and, in some cases, the *Alumni* and the honorary graduates are recorded together without distinction. All this can and should be rectified.

For most of the facts here recorded, I am indebted to the DENTAL REGISTER. Though not emanating from, nor the organ of the College, it has ever been faithful to its interests; and till within the last year, it has always had one or more of the Faculty on its editorial staff. It has been a faithful ally of our *Alma Mater*, and an impartial mouthpiece for her *Alumni*.

"Finally, Brethren, Farewell."

## Figures and Tables

**Figure f1:**